# Socioeconomic Differences in Cigarette Smoking Among Sociodemographic Groups

**DOI:** 10.5888/pcd16.180553

**Published:** 2019-06-13

**Authors:** Bridgette E. Garrett, Brandi N. Martell, Ralph S. Caraballo, Brian A. King

**Affiliations:** 1 Office on Smoking and Health, National Center for Chronic Disease Prevention and Health Promotion, Centers for Disease Control and Prevention, Atlanta, Georgia

## Abstract

We examined variations in cigarette smoking by socioeconomic status (education and poverty status) in relation to population sociodemographic characteristics (age, race/ethnicity, region and sex). We analyzed data from a nationally representative sample of US adults by using combined data from the National Survey on Drug Use and Health (2011–2014). Low socioeconomic status was generally associated with increased cigarette smoking prevalence by age, race/ethnicity, and region, irrespective of sex. The only exceptions were for Asian and Hispanic women, where low educational attainment was not associated with a high prevalence of cigarette smoking, and among Hispanic men and Asian women, where there was no association between poverty status and smoking. Efforts to reach smokers of low socioeconomic status by using proven tobacco control strategies could reduce disparities in cigarette smoking and smoking-related disease and death.

**Summary**

**What is already known about this topic?**

Socioeconomic disparities in cigarette smoking continue to exist despite progress in reducing cigarette smoking in the general population and among certain demographic groups.

**What is added by this report?**

Few studies have assessed the extent to which cigarette smoking differs among sociodemographic groups relative to their socioeconomic status. Findings from this report demonstrate that US adults with low socioeconomic status generally have high cigarette smoking prevalence irrespective of the sociodemographic characteristics of the population.

**What are the implications for public health practice?**

Efforts to reach low socioeconomic smokers with proven tobacco control strategies could reduce disparities in cigarette smoking and smoking-related disease and death.

## Objective

Despite progress in reducing cigarette smoking in the general population, socioeconomic (eg, education, income) and sociodemographic characteristics (eg, age, sex, race/ethnicity, US region) of the US population continue to influence cigarette smoking prevalence and associated disparities in cigarette smoking (1). Low socioeconomic status is associated with large disparities in cigarette smoking. (2,3). Because socioeconomic status is a strong determinant of cigarette smoking (2,4), it may also influence sociodemographic disparities in cigarette smoking. Few studies have assessed the extent to which cigarette smoking differs among sociodemographic groups relative to socioeconomic status. We assessed the relationship between socioeconomic status and sociodemographic characteristics as it relates to disparities in cigarette smoking.

## Methods

We used data on cigarette smoking from the National Survey on Drug Use and Health (NSDUH), an annual household survey that collects data on substance use, including tobacco use, from a nationally representative sample of the US noninstitutionalized, civilian population aged 12 or older. The survey design, methods, and general parameters of NSDUH are described elsewhere (5). For this analysis, we combined annual data from NSDUH survey years 2011 to 2014 to obtain sufficient sample sizes to calculate estimates of current cigarette smoking for the assessed sociodemographic and socioeconomic subgroups of adults aged 18 or older (N = 188,673) and adults aged 25 or older (N = 114,759). The average, weighted response rate for NSDUH 2011–2014 was 60.58% for respondents aged 18 or older and 59.67% for respondents aged 25 or older.

For our study, current smoking was defined as smoking all or part of a cigarette within the 30 days preceding the interview. Low socioeconomic status was defined as having less than a high school diploma (adults aged ≥25) and living below the poverty threshold (adults aged ≥18), which we calculated as a percentage of the US Census Bureau’s poverty thresholds (federal poverty level). We assessed sociodemographic groups by age (18–24, 25–44, 45–64, and ≥65), race/ethnicity, and US region.

Weighted prevalence of current cigarette smoking was computed among men and women by educational attainment and by poverty status. Prevalence estimates were calculated with 95% confidence intervals. Differences in prevalence estimates were assessed among men and women by *t* test between levels of educational attainment and poverty status (*P* < .05 denoting significance). These analyses were conducted separately by age, by race/ethnicity, and by US region. We used SAS SUDAAN (RTI International) for analyses. Survey weights were used to account for different probabilities of selection and nonresponse.

## Results

**Cigarette smoking by education**. Overall, among both men and women, an inverse significant association between current cigarette smoking and education was observed, with higher smoking prevalence among people with lower educational attainment (Table 1). Current smoking prevalence was 31.6% among people with no high school diploma, 27.5% among those with a high school diploma, 25.1% among those with some college but no bachelor’s degree, and 10.8% among those with a bachelor’s degree.

**Table 1:** Current Cigarette Use Among US Adults Aged 25 or Older, by Sociodemographic Characteristic and Education Level, National Survey on Drug Use and Health, 2011–2014^a^

Characteristic	Less Than High School Diploma [Reference], % (95% CI)	High School Diploma, % (95% CI) [*P* Value]	Some College (No Degree), % (95% CI) [*P* Value]	College Graduate, % (95% CI) [*P* Value]
**Overall^b^**	31.6 (30.6–32.7)	27.5 (26.8–28.2) [<.001]	25.1 (24.4–25.8) [<.001]	10.8 (1.4–11.3) [<.001]
**Age, y**				
25–44	42.8 (41.2–44.3)	37.0 (36.0–38.1) [<.001]	31.5 (3.6–32.4) [<.001]	14.6 (14.0–15.3) [<.001]
45–64	35.0 (33.1–36.9)	28.8 (27.6–3.0) [<.001]	24.4 (23.3–25.7) [<.001]	9.4 (8.6–1.2) [<.001]
≥65	12.8 (11.2–14.6)	11.3 (1.2–12.5) [.16]	11.2 (9.9–12.7) [.17]	5.0 (4.1–6.1) [<.001]
**Race/ethnicity**				
Non-Hispanic white	41.6 (39.9–43.3)	28.8 (28.0–29.6) [<.001]	26.2 (25.4–27.1) [<.001]	11.3 (1.7–11.8) [<.001]
Non-Hispanic black	37.0 (33.9–40.1)	29.2 (27.3–31.1) [<.001]	25.0 (23.1–27.0) [<.001]	8.9 (7.6–1.4) [<.001]
Non-Hispanic Asian	12.5 (8.1–18.7)	9.8 (7.3–13.2) [.39]	14.4 (11.6–17.7) [.53]	7.4 (6.1–8.9) [.06]
Hispanic	17.2 (15.8–18.7)	2.4 (18.7–22.3) [.005]	19.9 (18.2–21.8) [.02]	12.1 (1.4–14.0) [<.001]
**US Census region^c^**				
Northeast	31.2 (28.6–34.0)	27.0 (25.4–28.6) [.008]	25.4 (23.8–27.2) [<.001]	11.1 (1.1–12.1) [<.001]
Midwest	37.4 (35.2–39.8)	3.6 (29.3–31.9) [<.001]	27.7 (26.4–29.0) [<.001]	11.6 (1.8–12.4) [<.001]
South	33.4 (31.7–35.1)	28.0 (26.8–29.2) [<.001]	25.9 (24.7–27.1) [<.001]	11.7 (1.9–12.5) [<.001]
West	24.5 (22.6–26.6)	23.2 (21.7–24.8) [.29]	21.3 (19.8–22.8) [.009]	8.9 (8.0–9.9) [<.001]

a Source: Substance Abuse and Mental Health Services Administration, Center or Behavioral Health Statistics and Quality, National Survey on Drug Use and Health; surveys for 2011–2014 (8–11).

b Overall row includes data on respondents who reported being of more than one racial/ethnic group although these data were excluded from numbers in race/ethnicity categories.

c Northeast: Connecticut, Maine, Massachusetts, New Jersey, New Hampshire, New York, Pennsylvania, Rhode Island, and Vermont; Midwest: Illinois, Indiana, Iowa, Kansas, Michigan, Minnesota, Missouri, Nebraska, North Dakota, Ohio, South Dakota, and Wisconsin; South: Alabama, Arkansas, Delaware, District of Columbia, Florida, Georgia, Kentucky, Louisiana, Maryland, Mississippi, North Carolina, Oklahoma, South Carolina, Tennessee, Texas, Virginia, and West Virginia; West: Alaska, Arizona, California, Colorado, Hawaii, Idaho, Montana, Nevada, New Mexico, Oregon, Utah, Washington, and Wyoming.

Some sociodemographic differences were observed by age, race/ethnicity, and US region. By age group, smoking was generally highest in the youngest age group (25–44) with less than a high school diploma among both men and women (Table 2). Smoking prevalence was higher among white and black men with or without a high school diploma than among Hispanic adult men with similar educational attainment. Smoking prevalence was higher among adult white and black women with or without a high school diploma than among Asian and Hispanic adult women with similar education. Smoking prevalence was lowest among men and women living in the West, including those with low education levels.

**Table 2:** Current Cigarette Smoking Among US Adults Aged 25 or Older, by Sex and Education Level, National Survey on Drug Use and Health, 2011–2014^a^

Characteristic	Men				Women			
	Less Than High School Diploma [Reference], % (95% CI)	High School Diploma, % (95% CI) [*P* Value]	Some College (No Degree), % (95% CI) [*P* Value]	College Graduate, % (95% CI) [*P* Value]	Less Than High School Diploma [Reference], % (95% CI) [*P* Value]	High School Diploma, % (95% CI) [*P* Value]	Some College (No Degree), % (95% CI) [*P* Value]	College Graduate, % (95% CI) [*P* Value]
**Overall^b^**	36.7 (35.2–38.3)	3.7 (29.7–31.7) [<.001]	26.8 (25.7–27.9) [<.001]	11.7 (11.0–12.4) [<.001]	26.3 (24.9–27.8)	24.6 (23.7–25.5) [.04]	23.7 (22.9–24.6) [<.001]	1.1 (9.5–1.6) [<.001]
**Age, y**								
25–44	47.6 (45.5–49.8)	39.9 (38.4–41.3) [<.001]	33.3 (31.8–34.7) [<.001]	17.7 (16.7–18.9) [<.001]	36.7 (34.5–38.9)	33.8 (32.4–35.2) [.02]	3.0 (28.8–31.2) [<.001]	12.0 (11.3–12.8) [<.001]
45–64	38.5 (35.7–41.3)	3.0 (28.4–31.8) [<.001]	25.5 (23.7–27.5) [<.001]	8.8 (7.8–9.9) [<.001]	31.1 (28.6–33.7)	27.6 (26.1–29.2) [.02]	23.5 (22.1–25.0) [<.001]	9.9 (8.9–11.1) [<.001]
≥65	15.5 (12.8–18.6)	13.1 (11.2–15.3) [.17]	12.0 (9.7–14.8) [.07]	5.5 (4.2–7.2) [<.001]	1.7 (8.9–12.8)	1.2 (8.9–11.7) [.70]	1.7 (9.1–12.5) [.99]	4.3 (3.3–5.7) [<.001]
**Race/ ethnicity**								
Non-Hispanic white	45.0 (42.7–47.2)	31.1 (3.0–32.3) [<.001]	26.9 (25.6–28.2) [<.001]	11.5 (1.8–12.2) [<.001]	37.9 (35.6–4.2)	26.6 (25.6–27.7) [<.001]	25.7 (24.7–26.8) [<.001]	11.1 (1.4–11.8) [<.001]
Non-Hispanic black	44.8 (40.1–49.6)	35.1 (32.3–38.1) [<.001]	28.3 (25.2–31.6) [<.001]	9.8 (7.8–12.3) [<.001]	29.5 (25.8–33.5)	23.8 (21.4–26.3) [.01]	22.7 (2.4–25.2) [.004]	8.2 (6.6–1.3) [<.001]
Non-Hispanic Asian	—c	—c	—c	—c	6.0 (3.0–11.8)	6.4 (3.8–1.4) [.89]	8.6 (6.0–12.3) [.33]	3.4 (2.3–5.0) [.25]
Hispanic	22.6 (20.3–25.0)	25.3 (22.7–28.1) [.13]	23.6 (2.7–26.7) [.58]	14.0 (11.4–17.0) [<.001]	11.8 (1.1–13.7)	15.5 (13.3–17.9) [.01]	16.6 (14.5–18.9) [<.001]	1.3 (8.3–12.6) [.29]
**US Census region^d^**								
Northeast	35.2 (31.5–39.0)	29.6 (27.4–31.9) [.01]	26.6 (24.1–29.3) [<.001	12.1 (1.8–13.7) [<.001]	27.3 (23.8–31.2)	24.5 (22.4–26.8) [.20]	24.5 (22.4–26.7) [.20]	1.0 (8.9–11.4) [<.001]
Midwest	42.0 (38.9–45.2)	32.8 (31.0–34.6) [<.001]	28.4 (26.5–3.4) [<.001]	12.1 (1.9–13.4) [<.001]	32.2 (29.3–35.3)	28.6 (26.9–3.4) [.04]	27.2 (25.6–28.8) [.004]	11.1 (1.1–12.2) [<.001]
South	38.3 (35.9–40.8)	31.5 (29.8–33.2) [<.001]	28.2 (26.4–3.1) [<.001]	12.5 (11.3–13.7) [<.001]	28.4 (26.2–3.6)	24.8 (23.3–26.3) [.008]	24.2 (22.7–25.7) [.002]	1.9 (9.9–12.1) [<.001]
West	30.9 (27.8–34.2)	27.5 (25.2–29.8) [.09]	23.6 (21.3–26.0) [<.001]	9.9 (8.5–11.4) [<.001]	18.0 (15.7–2.6)	19.3 (17.5–21.3) [.39]	19.2 (17.5–21.0) [.44]	8.0 (6.9–9.2) [<.001]

a Source: Substance Abuse and Mental Health Services Administration, Center or Behavioral Health Statistics and Quality, National Survey on Drug Use and Health; surveys for 2011–2014 (8–11).

b Overall row includes data on respondents who reported being of more than one racial or ethnic group although these data were excluded from numbers in race/ethnicity categories.

c Low precision; no estimate reported.

d Northeast: Connecticut, Maine, Massachusetts, New Jersey, New Hampshire, New York, Pennsylvania, Rhode Island, and Vermont; Midwest: Illinois, Indiana, Iowa, Kansas, Michigan, Minnesota, Missouri, Nebraska, North Dakota, Ohio, South Dakota, and Wisconsin; South: Alabama, Arkansas, Delaware, District of Columbia, Florida, Georgia, Kentucky, Louisiana, Maryland, Mississippi, North Carolina, Oklahoma, South Carolina, Tennessee, Texas, Virginia, and West Virginia; West: Alaska, Arizona, California, Colorado, Hawaii, Idaho, Montana, Nevada, New Mexico, Oregon, Utah, Washington, and Wyoming.

**Cigarette smoking by poverty status**. Smoking prevalence overall was 41.1% among men with incomes below the federal poverty level and 23.7% among men with incomes at or above the poverty level (Table 3). Prevalence was 32.5% among women with incomes below the federal poverty level and 18.3% among those with incomes at or above the poverty level. Both men and women with incomes below the federal poverty level had a significantly higher smoking prevalence than those who lived at or above the poverty level, except for Asian women and Hispanic men. High smoking prevalence was observed among certain groups of men and women with incomes below the federal poverty level: white men (50.9%), white women (44.8%), black men (44.1%), American Indian/Alaska Native men (53.7%), and American Indian/Alaska Native women (49.0%).

**Table 3:** Current Cigarette Smoking Among US Adults Aged 18 or Older, By Sociodemographic Characteristics and Poverty Status^a^, National Survey on Drug Use and Health, 2011–2014^b^

Characteristic	Men		Women		Total	
	Below, % (95% CI)	At or Above, % (95% CI) [*P* Value]	Below, % (95% CI)	At or Above, % (95% CI) [*P* Value]	Below, % (95% CI)	At or Above, % (95% CI) [*P* Value]
**Overall^c^**	41.1 (39.8–42.5)	23.7 (23.2–24.2) [<.001]	32.5 (31.4–33.6)	18.3 (17.8–18.7) [<.001]	36.0 (35.1–36.9)	2.9 (2.6–21.3) [<.001]
**Age, y**						
18–24	38.9 (37.5–40.3)	35.4 (34.6–36.2) [<.001]	28.6 (27.6–29.7)	24.7 (24.0–25.4) [<.001]	33.2 (32.3–34.2)	3.3 (29.8–3.9) [<.001]
25–44	45.3 (43.2–47.5)	3.0 (29.3–3.8) [<.001]	35.9 (34.3–37.5)	22.2 (21.5–22.8) [<.001]	39.6 (38.3–41.0)	26.2 (25.6–26.7) [<.001]
45–64	43.4 (40.5–46.4)	2.7 (19.8–21.6) [<.001]	39.2 (36.6–41.9)	18.6 (17.8–19.4) [<.001]	41.0 (39.0–43.1)	19.6 (19.0–2.3) [<.001]
≥65	20.4 (14.8–27.4)	1.1 (9.1–11.2) [.002]	13.5 (1.8–16.8)	8.7 (7.9–9.5) [.002]	15.7 (13.1–18.8)	9.3 (8.6–1.0) [<.001]
**Race/ethnicity**						
Non-Hispanic white	50.9 (48.9–52.9)	23.7 (23.2–24.3) [<.001]	44.8 (43.1–46.5)	2.2 (19.7–2.8) [<.001]	47.4 (46.1–48.7)	22.0 (21.5–22.4) [<.001]
Non-Hispanic black	44.1 (41.2–47.1)	25.9 (24.3–27.5) [<.001]	3.9 (28.6–33.3)	15.8 (14.6–17.1) [<.001]	35.9 (34.1–37.8)	2.6 (19.6–21.7) [<.001]
American Indian/Alaska Native	53.7 (43.7–63.4)	35.7 (29.1–42.9) [.004]	49.0 (4.2–57.8)	31.7 (26.1–37.9) [<.001]	5.8 (43.6–58.0)	33.7 (29.3–38.5) [<.001]
Non-Hispanic Asian	24.2 (18.4–31.0)	14.5 (12.7–16.6) [.004]	7.6 (5.3–1.7)	5.7 (4.7–7.0) [.22]	15.0 (12.0–18.7)	9.8 (8.7–1.9) [.004]
Hispanic	25.5 (23.3–27.8)	23.5 (22.3–24.8) [.14]	16.8 (15.2–18.6)	13.1 (12.0–14.2) [<.001]	2.4 (19.1–21.8)	18.6 (17.8–19.5) [.02]
**US Census region^d^**						
Northeast	39.6 (36.4–42.9)	31.5 (29.8–33.2) [<.001]	31.2 (28.6–34.0)	18.4 (17.4–19.4) [<.001]	34.4 (32.5–36.5)	2.4 (19.6–21.2) [<.001]
Midwest	49.1 (46.3–51.8)	25.5 (24.6–26.4) [<.001]	41.3 (39.0–43.7)	21.1 (2.4–21.9) [<.001]	44.6 (42.8–46.4)	23.3 (22.6–23.9) [<.001]
South	43.2 (41.0–45.4)	25.0 (24.2–25.8) [<.001]	32.9 (31.2–34.6)	19.1 (18.3–19.9) [<.001]	37.1 (35.7–38.5)	22.0 (21.4–22.6) [<.001]
West	32.3 (29.7–35.0)	21.0 (19.9–22.1) [<.001]	25.4 (23.4–27.6)	14.1 (13.2–15.0) [<.001]	28.4 (26.7–3.1)	17.5 (16.8–18.3) [<.001]

a Household income in relationship to the federal poverty level.

b Source: Substance Abuse and Mental Health Services Administration, Center or Behavioral Health Statistics and Quality, National Survey on Drug Use and Health; surveys for 2011–2014 (8–11).

c Overall row includes data on respondents who reported being of more than one racial or ethnic group although these data were excluded from numbers in race/ethnicity categories.

d Northeast: Connecticut, Maine, Massachusetts, New Jersey, New Hampshire, New York, Pennsylvania, Rhode Island, and Vermont; Midwest: Illinois, Indiana, Iowa, Kansas, Michigan, Minnesota, Missouri, Nebraska, North Dakota, Ohio, South Dakota, and Wisconsin; South: Alabama, Arkansas, Delaware, District of Columbia, Florida, Georgia, Kentucky, Louisiana, Maryland, Mississippi, North Carolina, Oklahoma, South Carolina, Tennessee, Texas, Virginia, and West Virginia; West: Alaska, Arizona, California, Colorado, Hawaii, Idaho, Montana, Nevada, New Mexico, Oregon, Utah, Washington, and Wyoming.

## Discussion

Our study showed that low socioeconomic status is generally associated with a high prevalence of cigarette smoking by age, race/ethnicity, and US region. Moreover, these associations were generally consistent across sexes. The only exceptions were for Asian and Hispanic women, where lower education was not associated with higher cigarette smoking, and for Hispanic men and Asian women, where we saw no differences by poverty status.

Because both sociodemographic characteristics and socioeconomic status can influence cigarette smoking behavior (2,4), an understanding is needed of how these factors contribute to cigarette smoking disparities and associated health outcomes. For example, cigarette smoking among American Indian/Alaska Native men who lived at or above the federal poverty level was higher than among white men who lived at or above the poverty level (35.7% vs 23.7%). However, prevalence was similar among both groups who lived below the poverty level. Although an educational gradient in smoking was observed among Hispanic men, no differences were observed among them by poverty level. These findings are consistent with previous research showing that income gradients in cigarette smoking are observed primarily among non-Hispanic whites and blacks, but are less evident among Hispanics (2,6,7).

This study had limitations. Data were self-reported and were not biochemically validated. Because of small sample sizes for certain subgroups, such as American Indians/Alaska Natives and Asian men by educational attainment, estimates were not reported for these groups. Finally, this study only included persons in aggregate racial/ethnic populations, that is, cigarette smoking prevalence among subgroups of the broader racial/ethnic population categories were not reported.

In conclusion, these findings demonstrate that US adults with low socioeconomic status generally have high prevalence of cigarette smoking in relationship to various sociodemographic characteristics, irrespective of sex. Because disparities in tobacco use involve a complex interplay of demographic, social, and economic factors across the life course, comprehensive tobacco control efforts that consider social and economic contexts are important to advance progress in reducing cigarette smoking in socioeconomically disadvantaged populations.
